# Preventing Opioid Use Disorders among Fishing Industry Workers

**DOI:** 10.3390/ijerph15040648

**Published:** 2018-03-31

**Authors:** Angela Wangari Walter, Cesar Morocho, Lauren King, John Bartlett, Debra Kelsey, Monica DeSousa, Gretchen Biesecker, Laura Punnett

**Affiliations:** 1 Department of Public Health, University of Massachusetts Lowell, Lowell, MA 01854, USA; cesar_morocho@uml.edu; 2 Fishing Partnership Support Services; Burlington, MA 01803, USA; lking@fishingpartnership.org (L.K.); jbartlett@fishingpartnership.org (J.B.J.); dkelsey@fishingpartnership.org (D.K.); mdesousa@fishingpartnership.org (M.D.); gbiesecker@fishingpartnership.org (G.B.); 3 Center for the Promotion of Health in the New England Workplace (CPH-NEW), University of Massachusetts Lowell, Lowell, MA 01854, USA; laura_punnett@uml.edu; 4 Department of Biomedical Engineering, University of Massachusetts Lowell, Lowell, MA 01854, USA

**Keywords:** opioid use disorders, prevention, industry workers, workplace health, community based participatory research

## Abstract

Fishing industry workers are at high risk for work-related musculoskeletal disorders (MSDs) and injuries. Prescription opioids used to treat pain injuries may put these workers at increased risk for developing substance disorders. Using a Community-Based Participatory Research approach, formative research was conducted to inform the eventual development of relevant interventions to prevent and reduce opioid use disorders among fishing industry workers. Qualitative interviews (*n* = 21) were conducted to assess: knowledge and attitudes about opioid use disorders; features of fishing work that might affect use and/or access to treatment; and community and organizational capacity for prevention and treatment. Participants reported numerous pathways connecting commercial fishing with opioid use. The combination of high stress and physically tasking job duties requires comprehensive workplace interventions to prevent chronic pain and MSDs, in addition to tailored and culturally responsive treatment options to address opioid use disorders in this population. Public health programs must integrate workplace health and safety protection along with evidence-based primary, secondary, and tertiary interventions in order to address opioid use disorders, particularly among workers in strenuous jobs.

## 1. Introduction

Opioid use disorders have become a major public health concern across the U.S. Opioid-related drug overdoses are currently the leading cause of injury death in the U.S., surpassing motor vehicle accidents and deaths from firearms [1]. In 2016 alone, more than 63,600 people died in the U.S. of drug overdoses primarily from synthetic opioids such as fentanyl, fentanyl analogs, heroin, and opioid pain relievers [2,3]. Misuse of and addiction to prescription pain relievers, particularly the use of opioid analgesics including hydrocodone, oxycodone, oxymorphone, and others, has been documented as one of several pathways to opioid overdose [1,2,4]. Approximately 21 to 29 percent of individuals who are prescribed opioids for chronic pain in the U.S. misuse them [5], 8 to 12 percent of individuals who are prescribed opioids develop an opioid use disorder [5], and 4 to 6 percent of those who misuse prescription opioids transition to heroin [6,7,8]. Not only do opioid and other substance use disorders have a negative impact on individuals and their families, but they also affect the productivity of U.S. industries. Substance use has been linked to workplace injuries, as well as employee absenteeism and turnover [9,10,11,12,13]. The link between opioid use and occupational injury is apparently bi-directional, as there is also evidence of an association between work-related injuries and opioid dependence [9,14,15].

Commercial fishing industry workers are at a high risk for injuries, illnesses, and fatalities [16,17]. Occupational fishing requires long work schedules averaging 14.6 or more hours per day [18], consisting of hard physical labor, which can lead to musculoskeletal disorders (MSDs) and pain [19,20,21,22]. Among all workers, those with chronic pain are likely to receive opioid prescriptions [23,24], and the prescription of opioid pain relievers for treating moderate to severe pain is known to be associated with depression and subsequent opioid dependence [25,26,27,28]. Thus, it is plausible to anticipate that commercial fishing industry workers with MSDs, chronic pain, or traumatic injury who are using prescribed opioids are at high risk for developing an opioid use disorder.

In the U.S., the opioid epidemic has negatively impacted the Commonwealth of Massachusetts with increasing morbidity, mortality, poverty, and incarceration rates [29]. The age-adjusted rate of opioid overdose deaths per 100,000 people in Massachusetts is higher than the national average [30]. In 2016, Massachusetts reached the highest rate of opioid-related deaths ever at 30.9 deaths per 100,000 people [31], with a 3.5 fold increase in opioid-related deaths from 2010 to 2016 [31]. Since 2000, the number of opioids prescribed to Massachusetts residents has increased annually by approximately 7% [30]. In 2015, one out of every six Massachusetts residents received an opioid prescription from a health care provider, and on average, more than three of those prescriptions were filled [30]. Despite the rise in opioid-related prescriptions between 2015–2016, the percentage of overdose deaths attributed to fentanyl or heroin was higher than prescription opioid-related deaths [31]. However, it is important to note that while drugs such as fentanyl and heroin are more prevalent than other opioids among decedents’ cause of death, most decedents had filled an opioid prescription within four years of their death [32]. Evidence suggests that the misuse of prescription opioids may be an important pathway to the use of illegal drugs such as heroin [6,33,34]. 

In Massachusetts, the city of New Bedford is among the top five cities with the highest opioid overdose deaths between 2012–2016 [35]. The number of opioid-related deaths in New Bedford has more than doubled between 2012 and 2016, with 55 deaths in 2016 compared to 26 deaths in 2012 [35]. This is significant because New Bedford is one of the most valuable fishing ports in the U.S., with a high proportion of fishing industry workers and community members [35,36]. It is important to assess and develop interventions to prevent opioid use disorders focusing on the unique needs of commercial fishing industry workers, who are likely to receive opioid prescriptions because of injuries resulting from the nature of their work. Community-based interventions can increase community participation, ownership, and buy-in, which are all critical for success. 

This formative study used a community-based participatory research (CBPR) approach to investigate the ways in which commercial fishing might directly or indirectly influence the use of opioids and other pain medications as well as mood-altering substances. We also sought to characterize knowledge, attitudes, and perceptions about these substances in the fishing community and to document the scope and availability of prevention (primary, secondary, and tertiary), treatment, and recovery support services to meet the unique occupational needs of fishing industry workers. It is hoped that findings from this study will inform the eventual development of comprehensive and culturally relevant interventions to prevent and reduce opioid use disorders with a specific focus on fishing industry workers. 

## 2. Materials and Methods 

This study was conducted through a partnership between the University of Massachusetts Lowell (UML) and Fishing Partnership Support Services (FPSS). FPSS is a nonprofit organization that conducts initiatives to promote the health and well-being of commercial fishing workers and their families across New England. Through a coordinated CBPR approach, the UML and FPSS conducted formative research to assess: knowledge, attitudes, and perceptions of opioid use disorders; organizational capacity for prevention and treatment; and public health interventions that address the needs of this population. 

**Figure 1:**
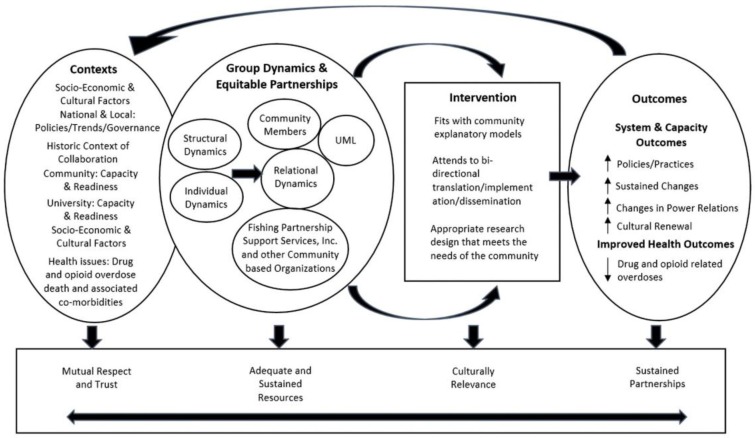
Community-based participatory research framework.

The CBPR framework comprises four dimensions: first, contextual factors in the community and the research organization(s) that shape the research project design; second, group dynamics within the community and in the community-researcher partnerships that influence the research design and the intervention; third, an intervention implementation process that reflects the first two factors as well as the specific initiative developed in the community; and fourth, the system, capacity, and health outcomes resulting from the initiative undertaken [37,38]. The conceptual framework in Figure 1 depicts how the CBPR process was intended to contribute to and guide the progression of research leading to interventions to improve health [37,38]. The project was reviewed and approved by the UML Institutional Review Board, Protocol No. 17-066.

In-depth key informant interviews were conducted with stakeholders representing local community members: commercial fishing industry workers, such as fishing boat owners, fishing boat captains, fishing crew members, and owners from businesses related to the fishing industry; systems of care, such as health department coordinators, educators, and physical and behavioral health providers; and the criminal justice system, such as prosecutors and parole officers. Based on previous work in New Bedford, FPSS developed a database with the contact information of 108 potential participants from the aforementioned professions. Staff from FPSS reached out to these participants and their networks when conducting outreach and recruiting study participants. Participants were recruited through face to face conversation, phone calls, and email communication. The interviews required about an hour, were conducted in English, and were audio-recorded. All participants completed a consent form and were given a $25 gift card in compensation for their time. 

A semi-structured interview guide was organized by key domains and used to facilitate interviews. The guide sought to: (1) assess knowledge, attitudes, and perceptions regarding the extent of opioid use disorders; (2) examine community and organizational capacity for preventing and treating opioid use disorders in the community; and (3) understand current public health initiatives or programs that would be needed to address substance use and misuse from the perspective of commercial fishing industry workers and community members. Top level interview questions from the interview guide can be found in Table 1.

Qualitative data were analyzed using NVivo 11 (QSR International Inc., Burlington, MA, USA) qualitative software [39]. Interviews were transcribed and exported into the NVivo database for line-by-line coding. The primary nodes consisted of the three domains in the structured interview guide. Major themes were structured into the principal aspects of each domain. Qualitative data were then sorted into secondary nodes representing minor themes within each domain. These minor themes were used to complement characteristics of the major themes while adding important details. Once thematic saturation was reached, themes and subthemes were catalogued into categories. Content and parameters of the data codes were refined and recorded. Responses were coded for intensity, frequency, and extensiveness by two authors independently (Angela Wangari Walter and Cesar Morocho). After completion of independent coding, both authors conducted a thorough revision of the codes and reconciled discrepancies. All coding discrepancies were discussed and resolved through coordinated discussions. Disguised verbatim accounts were recorded and analyzed. Quote excerpts and coding memos were developed according to themes. This rigorous analytic process was essential to ensure that the research was firmly grounded in the experiences of participants and provided a rich context in which to interpret the results.

## 3. Results

### 3.1. Descriptive Characteristics of Study Participants 

Twenty-one key informant interviews were conducted between June and August of 2017 in New Bedford, Massachusetts. Most of the participants were fishing industry (43%) and health care (33%) professionals (Table 2). The majority of the participants identified as male (67%), non-Hispanic (81%), White (81%), and having been born in the U.S. (71%). The mean age of study participants was 53 years. Most participants spoke more than one language; 38% spoke two languages and 38% reported speaking at least three languages. More than one-half of the participants reported speaking Portuguese (52%), which is consistent with the predominant Portuguese culture in New Bedford. Almost all participants were employed either full time (81%) or part time (14%). Most participants were college graduates (29%) or had some post graduate education (38%). 

### 3.2. Context Matters: Commercial Fishing and the Impact on Health 

Participants described commercial fishing as a very hard physical job, accompanied by multiple long work shifts, high pressures for economic productivity, and psychosocial job demands. They also stated that productivity and output are essential to being retained in the industry. Commercial fishing workers work long hours in order to meet productivity requirements within the constraints of current regulations. As these participants explained:

Quote 1: “There is no minimum wage, there is no hours regulation, no regulation for hours, sometimes you work eighteen hours a day, twenty hours a day, and (if) somebody don’t like it, don’t come back.”

Quote 2: “…they have put so much strict regulation for the fishermen in the National Oceanic and Atmospheric Administration (NOAA) that there is a lot more pressure on them than there was 10, 15, 20, 30 years ago. Now there are all these types of regulations that make their jobs hard and stresses them out to get a better income, to bring money back for their families.”

The nature of commercial fishing activities also profoundly affects families and social networks. Fishing industry interviewees reported being at sea without their families for extended periods of time, which may lead to anxiety or depression of workers and their family members, especially for families raising young children. 

Quote 3: “I’ve had families that you know, themselves are not struggling or didn’t struggle with addiction but their kids did. So, while their father was at the sea, you know the mother was the main you know provider at the house for support and these kids you know somehow got into alcohol, pills, heroin, cocaine and suddenly they had an addiction, and the mom was the only person that was there to help them. So, when the father was to come home, things were a lot more difficult to deal with, and because it’s still taboo in a lot of the culture in New Bedford about having you know a child or a loved one that suffers from an addiction.”

Further, there is an ongoing potential conflict between maintaining work productivity and the responsible use (or non-use) of both prescription and non-prescription opioids. Some key informants from the fishing community reported that substance use is important for maintaining enough strength and productivity to carry out strenuous work tasks. For instance, some fishing industry workers, particularly those who have chronic pain or MSDs, make arrangements to receive pain medication (including both legal and illegal opioids) in advance of a fishing trip, as a way to perform adequately. In addition, participants reported that industry workers involved in hard labor, such as those on scallop vessels, are more likely to be involved in substance use because the job demands in this sector are higher and require longer hours. 

Key informants from the fishing community specifically mentioned that younger commercial fishing workers tend to choose the scallop vessels because they are the most profitable. They discussed how young fishing workers who are not accustomed to the physical and emotional demands of commercial fishing may be at increased risk of chronic stress and eventual substance use and misuse.

Quote 4: “…sometimes you need a little puff or a little snort to get through the day, whatever it takes to get through the day, to make the money. These guys are working hard to make the money and everything else.”

Quote 5: “…in the last few years you see a lot of the men who work in these fishing boats because of the hard work, because of the long hours, you know having such many medical problems from back pain, breaking legs or whatever it is that, you know, they might need substance to address that issue...”

Quote 6: “…we have to start to educate the owners, and the captains that, the value of life should oversee and overwrite and overshadow performance and production to get that individual to get some help, they need to start to see this people as human beings, and not robots, not a machine that is just plug in and perform and making money for them.”

### 3.3. Qualitative Findings by Domain of the Interview Guide

#### 3.3.1. Knowledge, Attitudes, and Perceptions of Opioids and Other Drug Use Disorders in the Community 

Participants from the public health and criminal justice sectors reported that the opioid epidemic is widespread in New Bedford, and that there has been a steady increase in the number of emergency department visits associated with opioid overdoses in the last few years. Substance use disorders were known of in all groups by race, age, socioeconomic position, neighborhoods, and profession. Among all participants, there was the perception that everyone in the community knows someone who has or had a substance use disorder. This suggests that substance use disorders have impacted community members across the life span.

Quote 7: “I think that recognizing that this is not just something that affects, you know, a certain demographic people, affects all, it crosses demographics, it crosses all kinds of life, races, and origins. So, it is something that it is widespread and it is going to take a concerted community wide effort.”

Quote 8: “The one thing we do realize is that this is across the board in our community so we are visiting people in housing developments. We are visiting people in some of the better neighborhoods with relapses, BMWs and Mercedes in the driveway.”

However, despite the high prevalence of opioid and other drug use disorders in this community, there is a lack of understanding about opioid addiction as a disease, which has resulted in the stigmatization of individuals with substance use disorders. Some key informants expressed that substance use disorders occur mainly among teenagers, “junkies,” individuals experiencing homelessness, and people of lower socioeconomic status. Among some participants, there was a perception that substance use disorders would never occur in their own families. The stigma and shame around substance use disorders is perceived by individuals, family members, and social network(s) and represents a significant barrier for individuals seeking treatment. Several participants expressed that substance use disorders were the result of moral choice and not a chronic disease. Furthermore, there is a perception that people who suffer from substance use disorders are “rewarded” by the number of programs developed to help them overcome their problem.

Quote 9: “Addiction is not a disease, no you make that choice, you make that initial choice to stick that needle on your own and take that first go. So, it’s choice… everything is handed to you, you are rewarded for being a drug addict. Something that is illegal.”

Quote 10: “Narcan brings the guy back and the next thing you know is that they are still on drugs. They bring him back and how many times is that gonna (going to) happen? They need to get if something happens the first time. They need to get to that guy. I don’t know that I wanna (want to) give them 25 chances of keep coming back.”

Community members also expressed that a major contributing factor to the opioid epidemic is the availability of opioid prescription drugs. Prescription opioids are readily available on the street and are sold for relatively more than other street drugs, such as heroin. As such, there is a market for prescription opioids whereby individuals are able to sell them for profit. Individuals seeking street value prescription opioids may not understand the risks associated with the mixing of medications, self-medication, prescription sharing, and illegal use of prescription drugs that might also have been modified. 

Quote 11: “If I am a drug addict, if one (prescription) pill is 85 dollars, right. I can do what, 18, 19 bags of heroin. If I am gonna (going to) take that one pill, it is gonna (going to) last for a few hours. If I have 10 bags of heroin, that’s ten times. I am saving money.”

#### 3.3.2. Community and Organizational Capacity for Preventing and Treating Opioid Use Disorders 

##### Community Education and Awareness

Respondents described several relevant initiatives in the town of New Bedford. The fishing vessels from this community are required to have at least one commercial fishing industry worker trained and certified in both first aid and cardiopulmonary resuscitation (CPR). There have been opioid overdose awareness sessions embedded in these training and certification courses. In addition, some boat captains and crew members have received training in defibrillator use and opioid overdose reversal with Naloxone. However, these trainings are not universal across fishing boats or among all commercial fishing industry workers. 

In addition, key informants from fishing and healthcare professions reported that Seven Hills Behavioral Health, an integrated health and human service agency in New Bedford, has provided opioid awareness and Naloxone/Narcan as part of FPSS’s first aid and CPR course. The training provided by Seven Hills has been tailored specifically to the fishing industry. Fishing industry workers are trained to recognize an overdose and trained in the use of Naloxone to prevent a fatal overdose.

Furthermore, while the fishing community is a close-knit community, some key informants reported that community members do not openly discuss opioid and other drug use disorders and the associated risks or consequences. Reticence to discuss substance use and its impact was reported as a barrier to the effectiveness of training initiatives. Interviewees mentioned that some boat owners or captains know about substance use and misuse among crew members, but they choose to ignore this because of their focus on productivity. In addition, there are some companies and boat owners that conduct drug tests with the crew members, but this practice is not mandatory.

Quote 12: “…if I think, even I suspect someone is on drugs but he is one that performs good, I take it, and there is also law no drugs on the boat… so for captains once we leave it’s better not to know. It’s sad but it’s better not to know coz once I see anyone who is smoking marijuana I have to terminate, bring him home, call coast guard, call police, how are we gonna (going to) do that?”

Quote 13: “…in the fishing industry, and like altogether there is a people who are functioning and going to work every day and you know using drugs… I mean there is like a lot of functioning addicts… I think specially a lot of them are younger guys who are scallopers… Then, I mean sometimes before they even get on to the next trip you know they’re borrowing money from other crew members and stuff like that.”

Quote 14: “So, I know some vessel owners required drug testing, and I know some vessel owners don’t because it’s like who are you going to find to catch on your boats if you are not, I mean like the problem is so bad in New Bedford that is almost like you don’t even wanna (want to) test the situation because it’s like sometimes you know I mean if you get rid of the crews that you have, coz they are using drugs, you might, you’ll lose everyone in your boats.”

Participants from the healthcare profession highlighted that several organizations have developed education programs geared towards educating children, young adults, and parents. The Nurses to Prevent Opioid Abuse (NPOA) implemented the nationally recognized Hidden in Plain Sight program for parents to learn how to identify clues from a teen’s bedroom that might indicate if their child was experimenting with drugs or alcohol. The Positive Action Against Chemical Addiction (PAACA), which has developed recovery support services and youth risk prevention programs, also partners with several organizations in the community to provide comprehensive services for individuals at risk for and with substance use disorders. 

Key informants mentioned that trainings around opioid disorders have also been targeted to specific professions and key segments of the population. For instance, through the Inter-Church city council, church leaders received education related to opioid awareness, suicide prevention, and mental health. In addition, Physicians to Prevent Opioid Abuse (PPOA) conducted education and training programs for physicians to keep them updated about the guidelines for the management of prescription drugs. In addition, the Council on Aging conducted presentations to older adults regarding the proper storage of medication and the use of medication lock boxes. 

##### Outreach Programs

Resources now available in the community include a drop-in center that provides information and other services to community members regarding opioid and other drug use disorders. In addition, a multi-disciplinary team consisting of a chaplain, a police officer, an advocate, a navigator from New Bedford FPSS who is a recovery coach, and a social worker or a licensed mental health counselor reaches out to individuals who have experienced an overdose. This team contacts individuals and their families within twenty-four to seventy-two hours of the event to provide knowledge about treatment and recovery services. During an outreach visit, there is training in the use of Naloxone, and a free Naloxone kit is provided to the individual(s) in the home.

Quote 15: “…the drop-in center that’s gonna (going to) get some people an opportunity to say I am going to get help. So that’s what we are looking to do. Just to continue to give people a non-judgmental opportunity to come and it’s not just the people that are struggling. When you take care of people or you take care of patients, it’s the family and the community as well.” 

Last, police departments within New Bedford and other neighboring cities and towns have created an alliance to facilitate outreach activities within and across jurisdictions. Police departments inform each other if a resident from one city overdoses in another jurisdiction. This collaboration allows police departments to work closely, not only with other jurisdictions, but also with residents in their own community.

##### Diversion Programs

The Law Enforcement Assisted Diversion (LEAD) program is intended to support individuals who have substance use disorders by diverting them to treatment instead of incarceration. The Bristol County Jail and House of Correction is working on care transition plans for justice-involved individuals to ensure that they are linked to the right services upon release. Treating them provides a unique opportunity to decrease substance use and reduce associated criminal behavior.

##### Therapeutic and Support Groups

There are several therapeutic and support groups in New Bedford. The Learn to Cope support group provides parents, family members, spouses, and caregivers with informational material, guest speakers, overdose education, and Narcan enrollment/training at weekly meetings. There are also other support groups such as Alcoholic Anonymous and Narcotics Anonymous that are accessible to individuals seeking recovery from substance use disorders. In addition, there is a confidential 12-step support and recovery program that is tailored to meet the unique professional needs of law enforcement individuals with substance use disorders and those in recovery.

##### Treatment

Key informants from the healthcare profession identified several organizations that provide treatment for substance use disorders in New Bedford. These include: Seven Hills Behavioral Health, High Point Treatment Center, CleanSlate Addiction Treatment Center, and AdCare. Inpatient, and outpatient treatment services are available including medication-assisted treatment. Among the innovations in the access to treatment, the Massachusetts Addiction Recovery Software (MARSI) allows individuals and organizations to look up the status of open beds in detox, clinical support services, and transitional support services in Massachusetts.

#### 3.3.3. Recommendations for Public Health Initiatives

As described above, formative findings demonstrate that there are several programs and initiatives currently in place to address substance use and misuse in New Bedford. However, participants emphasized that there is an urgent need to tailor these interventions to meet the needs of fishing industry workers.

##### Tailored Outreach and Support Systems

Despite the community and organizational capacity identified by stakeholders above, there should be tailored outreach and support systems for fishing industry workers. For instance, some participants suggested having recovery coaches who understand and address the needs of the fishing industry workers, and that these recovery coaches should be current or former fishing workers in order to be able to create a cultural bond with the fishing workers in recovery. It should be noted that FPSS took first steps in this area by training an FPSS navigator as a peer recovery coach and involving the navigator in collaborative outreach initiatives.

Quote 16: “I think that one of the things that would be important to have would be somebody that represent that community, their own… I just think when you talked about you know the fishing industry being very close, a lot of times you need that special person inside that could connect to the rest of the world.”

Quote 17: “…when you are going to help somebody that relationship would better suited with somebody who came from there, coz they overcame, that’s the testimony, that’s the story, that’s the way to show you this is real, I got you, I’ll be with you. Because if you walk with them, and support them, there is a likelihood of succeed versus ok get clean, and then back out.”

##### Increasing Access to Substance Use Treatment

Interviewees highlighted a gap in the access to substance use treatment, particularly the lack of adequate beds for those in need of residential treatment, which delays treatment for those in need of services. Additionally, participants expressed concern about the lack of transitional care and support services for individuals leaving treatment. It is important to note that although informants mentioned the need for substance use treatment facilities and improved organizational capacity for treatment and support services, many community members are resistant to having these facilities in their neighborhoods—the “Not In My Back Yard (NIMBY)” attitude.

Quote 18: “I think it’s kind of hard like getting into facilities sometimes, I think with people you kinda (kind of) need to catch them when they are willing to seek treatment I think a lot of the barriers is that once someone is ready to go, sometimes the treatment is like that they don’t have a bed open, they’ll say, let’s try the end of the week, let’s try next week… I think sometimes the availability of services like right away isn’t there.” 

Quote 19: “…sometimes the services aren’t immediate, sometimes they don’t get a bed right away, you know what I mean, so if they don’t get the bed right away when they are ready, then they are gonna (going to) just get high again.”

Quote 20: “We have a number of treatment providers. The issue is acute treatment services and transitional treatment services. So, the acute treatment or detox, inpatient detox you know it’s hard to find locally and then when a person comes out of detox there is like almost no transitional sort of assistance for them, which is a problem because what happens is that they come out of detox, they don’t have a good step down and then they wind up going back to using and then it’s like a cycle. Which I think it’s part of the reason why the detox is so congested.”

##### Education and Raising Awareness 

Raising awareness across the life course regarding the complexity of opioid and other drug disorders is critical to addressing the opioid epidemic. Participants highlighted the importance of engaging fishing industry workers, such as boat owners and captains, in the development and delivery of preventive messages. For example, the identification of a champion from the fishing industry is essential for advocating and supporting the development and implementation of relevant interventions for this population. 

Quote 21: “…it’s important that you meet everyone where they are at… if we want to get the docs on board for you know some new initiative, we are gonna (going to) go to one of the doctors find a physician champion and then have him or her go out and kind of recruit other because its best to have you know doctor to doctor, fisherman to fisherman, captain to captain.”

Several participants echoed the need for education and awareness prevention efforts, which include multiple stakeholders across different age groups and generations, and embrace the real-life histories of fishing industry families. Informants noted the importance of family- and community-based approaches towards prevention and treatment.

Quote 22: “…you have to target the families and the only way, fishermen are gonna (going to) change is pressure from the families and, realistically, you are not gonna (going to) cure everybody.”

Quote 23: “I think the peer involvement or the community that is affected should be part of developing the decision making.”

Quote 24: “…it’s not just the fishermen per se, you have to get to the community. It has to be a community-wide partnership in order for it to work.”

## 4. Discussion

This formative study generated an in-depth picture of a commercial fishing community in Massachusetts, which could inform the development of comprehensive and culturally relevant interventions to prevent and reduce opioid use disorders. The findings illustrate the complexity of physical and psychosocial hazards in commercial fishing work, consistent with previous evidence of strenuous tasks and a high risk of injuries, MSDs, and depression [16,17,20]. The physical demands reported in commercial fishing put industry workers at high risk for developing chronic pain and MSDs [20,21,22]. As a result, industry workers are likely to receive one or more prescriptions to address chronic pain and other MSDs. The combination of high stress and physically demanding commercial fishing jobs, coupled with the risk for mental illness and chronic pain, may lead to adverse health outcomes including opioid and other drug use disorders [11,12,13,16]. Primary interventions that prevent work-related injuries in the fishing industry have the potential to reduce MSDs and chronic pain, which could in turn reduce opioid use and misuse among fishing industry workers. However, to the best of our knowledge, little is known to date about the extent of substance use and misuse in the fishing industry. This formative study contributes to understanding factors that may lead to substance use among fishing industry workers. 

Study findings also identified an ongoing potential conflict between work productivity and the responsible use (or non-use) of both prescription and non-prescription opioids in the fishing industry. Employers’ emphasis on work productivity often creates time pressure and other dynamics that conflict with worker safety, well-being, and self-care [40,41,42,43,44]. This study highlights this well-known challenge of high job demands and worker health and the need for multi-pronged approaches to address worker health in high demanding jobs such as the fishing industry. As noted by a select number of key informants, some boat captains or boat owners seem indifferent to substance use and misuse on the job, as long as their employees can work effectively. Some individuals use substances to achieve better cognitive functioning or better physical performance [45,46,47,48]--an issue not limited to commercial fishing. For example, there are reports of occupational drug use among female entertainment workers in order to meet the physical demands of their work [49]. 

Analysis of key informants’ perceptions showed that the opioid epidemic extends beyond the fishing industry and is widespread in the larger community. These perceptions correlate with the steady growth in the number of opioid overdose deaths in New Bedford over the last four years [35]. However, despite an ongoing opioid epidemic that requires immediate action across the community, as noted above, some key informants’ comments revealed stigma and a lack of understanding about opioid addiction as a disease, which suggests a great need to educate the community. Stigma poses a significant barrier to accessing much needed services [50]; some research suggests that stigma influences treatment outcomes [50] and the well-being of individuals in recovery [51]. Findings show that there is certainly a greater need to educate community members about addiction as a chronic disease and the public health principle of harm reduction, in order to reduce stigma and encourage prevention and treatment-seeking behaviors in the community. 

The assessment of the current community and organizational capacity showed that there are several prevention and treatment initiatives currently taking place to address substance use disorders in the community. These efforts have traditionally focused on parents with children in high school, youth, and older adults. The diversity in target populations correlates with the scope of the opioid epidemic in this community [35]. In addition, there are a number of initiatives to support individuals in their process of recovery, such as support groups, treatment centers, diversion programs, and outreach programs.

Individuals with substance use disorders need access to evidence-based treatment. While the Affordable Care Act has expanded access to health insurance coverage, recent data show that only 59% of crew members in Northeast fisheries reported having health insurance coverage [18]. Many fishing industry workers may be underinsured or lack health insurance coverage altogether; and their fluctuating incomes may influence whether or not they purchase private insurance in the health insurance marketplace. The lack of health insurance coverage and underinsurance makes it difficult for individuals to get much needed addiction and or alternative pain management treatments. Ensuring that fishing industry workers have comprehensive health insurance coverage is critical. 

Moreover, complex fishing schedules affect the addiction recovery process for many commercial fishing industry workers seeking treatment. The approaches to substance use treatment are not necessarily in alignment with the fishing occupation. For example, substance use treatment might require residential stays for an extended period of time. Commercial fishing industry workers may not be able to initiate and stay in residential treatment because of their constant fishing trips, sometimes over multiple days, and the occupation’s transient nature. Further, residential treatment may pose an economic hardship for the fishing industry workforce that relies on seasonal employment.

Additionally, key informants highlighted a gap in the availability of residential treatment and transitional support services in the community, which is consistent with the lack of treatment beds and transitional supports in Massachusetts [52]. Transitional support services are key to the process of recovery because they provide a safe and controlled setting after the detoxification process [53]. For fishing industry workers with MSDs, comprehensive pain management initiatives that are safe and effective should be an integral part of the prevention and treatment initiatives to mitigate the risks of opioid use and dependence [54,55,56]. These efforts should include effective non-opioid pain management strategies to curtail the risk of opioid dependence and prevent opioid-related deaths. 

Key informants suggested the importance of involving multiple stakeholders in order to develop education and awareness initiatives that address opioid use in the fishing community. This recommendation is in alignment with the development of interventions anchored in the CBPR framework, which embraces a collaborative approach with community stakeholders to address locally relevant health issues [37,38,57]. Community interventions include: helping fishing families sign up for health care coverage, so they can access preventive services and treatment; providing ergonomic training to reduce injuries among fishing industry workers; conducting opioid awareness trainings as part of every safety and survival program; and connecting fishing industry workers with therapeutic support after they experience traumatic events. Community-driven initiatives compose a broad approach to address the opioid epidemic, with origins that extend beyond opioid analgesics and involve inequities in social determinants of health [58]. The education and awareness messages should not be limited to fishing workers, and they should reach the workers’ family in order to avoid intergenerational drug use. Family-based approaches have received empirical support addressing substance use disorders and suggest promising treatment outcomes [59]. Moreover, the engagement of a champion from the fishing community is key to spreading messages and tailoring them to the culture of the community. The champion might boost the empowerment of fishing organizations and unions to address opioid use disorders in the community.

This study has some limitations worth noting. This study used a convenience sample of stakeholders so the findings may not be generalizable. Participants were a self-selected group of stakeholders who may have been more interested in and/or more knowledgeable about opioid use than other community members. Future research should systematically recruit a larger sample of fishing industry workers and their family members to determine if their knowledge, perceptions, and attitudes are different or more varied.

As a strength, it should be noted that most of the fishing industry participants had been in the fishing profession for many years, which allowed them to speak about trends they had observed as well as their roles in different activities, job duties, and the realities that coexist in the different fisheries. Further, findings from this qualitative study are in alignment with previous research and anecdotal information on the extent of the opioid epidemic in New Bedford. For example, FPSS Navigators have been hearing similar reports about the opioid epidemic while providing community health interventions to fishing families. 

## 5. Conclusions

Given the current opioid epidemic in the U.S., any opportunity for primary or secondary prevention is vital to identify and actualize. This formative study provided new insights about some underlying drivers that may lead commercial fishers to substance use disorders. Commercial fishing industry workers are at high risk of opioid use disorders as a result of the physical and psychosocial hazards that typify the fishing occupation. The contextual factors that surround the fishing occupation also contribute to commercial fishing industry workers being at high risk of substance use and misuse. Primary, secondary, and tertiary interventions to address substance use disorders should consider the nature of the fishing occupation and the multiple underlying stressors that lead fishing workers to use alcohol and other drugs, including opioids. 

This study also identified an ongoing conflict between workplace culture, work productivity, and the responsible use (or non-use) of opioids. Given this conflict and the extent of the opioid epidemic, integrated and comprehensive initiatives should involve a community-wide effort to engage multiple stakeholders, especially considering the unique characteristics of the fishing community. Our study recognizes that community-informed interventions are central to shifting social norms, increasing safety practices, altering behavior, and promoting environmental change within the fishing workforce. Last, this study moves beyond the traditional health care paradigms by integrating behavioral health, workplace, and worker health fields to address opioid use and misuse. We encourage similar efforts in other communities to examine the potential contribution of working conditions to opioid use, as well as whether the available resources in the community are able to address those root causes in designing suitable primary and secondary prevention measures. 

## Figures and Tables

**Table 1:** Top level questions from the semi-structured interview guide.

Domain	Top Level Interview Questions
Knowledge, attitudes, and perceptions	In your opinion, what are the major reasons for substance use and abuse in this community?
Community and organizational capacity for preventing and treating opioid use disorders	To the best of your knowledge: what are the gaps in prevention and treatment services for individuals and families at risk for or living with substance use problems in this community?
Current public health initiatives or programs that would be needed	In your opinion, what is the most important strategy that should be considered to help people in this community to prevent substance use?

**Table 2:** Descriptive characteristics of study participants (*n* = 21).

Demographic Characteristic	*n* (%) or Mean (SD)
*Gender*	
Male	14 (66.7%)
Female	7 (33.3%)
*Age*	52.95 (SD 14.73)
*Birthplace*	
United States	15 (71.4%)
Other	6 (28.6%)
*Ethnicity*	
Hispanic or Latino/a	4 (19.0%)
Not Hispanic or Latino/a	17 (81.0%)
*Race ^1^*	
White	17 (81.0%)
More than one race	1 (4.8%)
Other	3 (14.3%)
*Highest level of education*	
Less than high school	1 (4.8%)
High school graduated	2 (9.5%)
Some college, no four-year degree	4 (19.0%)
College graduate	6 (28.6%)
Post-graduate or professional schooling after college	8 (38.1%)
*Profession*	
Fishing	9 (42.8%)
Criminal Justice	1 (4.8%)
Legislative	1 (4.8%)
Healthcare	7 (33.3%)
Educators	2 (9.5%)
Non-profit	1 (4.8%)

^1^ No participants self-identified as American Indian/Alaska Native, Native Hawaiian or Other Pacific Islander, or Black/African-American.
